# Supplementary material for: A data driven approach to address missing data in the 1970 British birth cohort
Source: BMC Med Res Methodol. 2026 Mar 26;26:91. doi: 10.1186/s12874-026-02833-0 (PMC13104459; doi:10.1186/s12874-026-02833-0)
Supplement: Supplementary file 1 — Supplementary Material 1. [file 12874_2026_2833_MOESM1_ESM.docx]

**APPENDIX: A data driven approach to address missing data in the 1970 British birth cohort**

**Section 1: Description of the 3 stages and predictors of non-response**

In Figure S1 we present how we identified the predictors of non-response for each sweep. We followed a 3-stage analytic approach, starting from 21021 variables. We then excluded “routed” variables (questions that depend on a specific response to a previous question), excluded variables with >40% missing, recoded variables with categories including <1% of cases if possible, and used index/score variables that combined information from many variables rather the individual constituent items wherever possible. We then used the remaining eligible variables as input (see Figure S1). At stage 3, we recoded some variables so that we do not face problems due to non-convergence of the imputation model (see Section 2, Tables S12-S20). At each stage we modelled non-response with a Poisson model with robust standard errors (modified Poisson regression).

Below we will show how to estimate relative risk by using the Poisson regression model with a robust error variance. Poisson regression is typically considered an effective method for analysing events when subjects are observed during the follow-up period (count data). However, when applied to binomial data, Poisson regression tends to overestimate the error in the estimated relative risk[1]. This issue can be corrected by using a robust error variance technique known as sandwich estimation[2], which leads to what is referred to as modified Poisson regression.

For example, let x_i_ (where i = 1, 2, …, n) represent a binary exposure (1 for exposed subjects and 0 for unexposed subjects). Moreover, the data can be summarized in the following 2-by-2 Table S1, from which we derived our notation.

***Table S1****: 2-by-2 of our data from which we derived our notation from the modified Poisson model*

|  | y = 1 (event) | y = 0 (no event) | Total |
| --- | --- | --- | --- |
| x = 1 (exposed) | a | b | n_1_ = a + b |
| x = 0 (unexposed) | c | d | n_0_ = c + d |
|  |  |  | n = n_1_ + n_0_ |

Assume that each subject i has an underlying risk that depends on x_i_, denoted as π(x_i_). The logarithmic link function is a suitable choice for modelling π(x_i_), leading to the equation log[π(x_i_)] = α + βx_i_. The relative risk (RR) is then represented by e^β^. If a Poisson distribution is assumed for y_i_, the log-likelihood function is given by:

$$l\left( \alpha,\beta\right)=C\sum_{i=1}^{n} \left[ y_{i}\left( \alpha+\beta x_{i} \right)+e^{\left( \alpha+\beta x_{i} \right)} \right]$$

where C is a constant, see[3]. After applying standard likelihood theory[3], we have that $e^{\left( \hat{\alpha} \right)}=\frac{C}{n_{0}}$ , ${\hat{\mathrm{RR}}=e}^{\left( \hat{\beta} \right)}=\frac{\alpha n_{0}}{C n_{1}}$ with the estimated variance being $\hat{\mathrm{var}}$($\hat{\mathrm{RR}}$)= $\frac{1}{\alpha}+\frac{1}{C}$

However, the error term is misspecified when the underlying data are binomially distributed. So, in this case, the sandwich estimator is used to make the appropriate correction. Specifically, the corrected variance is given by

$$var\left( \hat{\mathrm{RR}} \right)=\frac{1}{\alpha^{2}}\sum_{i=1}^{n_{1}} \left[ y_{i}-e^{\left( \alpha+\beta\right)} \right]^{2}+\frac{1}{C^{2}}\sum_{i=1}^{n_{0}} \left[ y_{i}-e^{\left( \alpha\right)} \right]^{2}$$

which is consistently estimated by $\hat{\mathrm{var}}$($\hat{\mathrm{RR}}$)= $\frac{1}{\alpha}-\frac{1}{n_{1}}+\frac{1}{C}-\frac{1}{n_{0}}$

This estimator is identical to the traditional variance estimator derived by using the delta method and an extension of this result can also incorporate covariates adjustment[4]. For more details, see[3].

Below, we describe our 3-stage procedure for non-response at sweep t:

**Stage 1**: We used univariable modified Poisson regressions of non-response at sweep t on each potential predictor of non-response at sweep 1 up to sweep t –1 in complete case analysis. We kept predictors with p-value<0.001.

**Stage 2**: We used multivariable modified Poisson regressions of non-response at sweep t on all predictors retained from stage 1, separately from sweep 1 up to sweep t –1. Keep predictors with p-value<0.05.

**Stage 3:** 4 variables (Sex, country of birth, participation in all previous sweeps and father’s socioeconomic status were used directly in Stage 3), were utilised directly in stage 3. These variables have been considered very strong predictors of non-response from our work in NCDS[5] and from other survey studies. At this stage (stage 3), we performed multiple imputation (MI) using all retained variables plus non-response at sweep t in the imputation model. MI multivariable modified Poisson regressions for all retained predictors at sweep 1, up to sweep t–1, adjusted for predictors at all previous (but not subsequent) sweeps. We kept predictors with p-value <0.001.

The main difference from the analytic strategy documented for NCDS[5] is that the approach for BCS70 was somewhat stricter (e.g. at Stage 1 the critical p-value was 0.001 versus 0.05 in NCDS, variables excluded from the list of potential predictors of non-response if they had missing data in >40% of the observations versus >50% in NCDS). For more details, see section 4 and Table S21.

In Table S2, we present in detail the number of variables selected in Stage 1, Stage 2 and Stage 3 for predictors of non-response at each sweep from the previous sweeps and in Tables S3-S11, we present the results from all the predictors of non-response after Stage 3, along with the estimated risk ratios and 95% confidence intervals.

***Figure S1****: Flowchart presenting the number of variables used to find the predictors of non-response*

**Number of available variables: N=21021**

Excluded “routed” variables (questions that depend on a specific response to a previous question), variables with >40% missing, variables with categories including >98% of cases, recoded variables with categories <1% if possible, excluded all binary variables with prevalence <1% of cases, and used index/score variables that combined information from many variables rather the individual constituent items

**Variables to be used for Stage 1-3: N=967**

Application of the Stage 1-3 process

**Variables to be used after Stage 3:**

**Vary from N=7 (at sweep 2) to**

**N=16 (at sweep 10)**

|  | **NR 2** | **NR 3** | **NR 4** | **NR 5** | **NR 6** | **NR 7** | **NR 8** | **NR 9** | **NR 10** |
| --- | --- | --- | --- | --- | --- | --- | --- | --- | --- |
| Wave 1 *Variables: 67* | S1: 28 S2: 15 | S1: 26 S2: 14 | S1: 30 S2: 13 | S1: 37 S2: 15 | S1: 30 S2: 14 | S1: 34 S2: 13 | S1: 35 S2: 13 | S1: 31 S2: 12 | S1: 35 S2: 11 |
| Wave 2 *Variables: 91* |  | S1: 15 S2: 8 | S1: 39 S2: 9 | S1: 56 S2: 6 | S1: 49 S2: 9 | S1: 55 S2: 11 | S1: 57 S2: 7 | S1: 49 S2: 8 | S1: 51 S2: 9 |
| Wave 3 *Variables: 162* |  |  | S1: 64 S2: 15 | S1: 88 S2: 14 | S1: 84 S2: 12 | S1: 91 S2: 13 | S1: 90 S2: 13 | S1: 82 S2: 9 | S1: 78 S2: 9 |
| Wave 4 *Variables: 108* |  |  |  | S1: 63 S2: 11 | S1: 39 S2: 7 | S1: 57 S2: 9 | S1: 65 S2: 12 | S1: 49 S2: 9 | S1: 54 S2: 7 |
| Wave 5 *Variables: 137* |  |  |  |  | S1: 35 S2: 7 | S1: 54 S2: 8 | S1: 64 S2: 13 | S1: 49 S2: 5 | S1: 53 S2: 10 |
| Wave 6 *Variables: 125* |  |  |  |  |  | S1: 56 S2: 11 | S1: 75 S2: 11 | S1: 56 S2: 12 | S1: 64 S2: 15 |
| Wave 7 *Variables: 148* |  |  |  |  |  |  | S1: 80 S2: 10 | S1: 69 S2: 17 | S1: 77 S2: 16 |
| Wave 8 *Variables: 51* |  |  |  |  |  |  |  | S1: 23 S2: 10 | S1: 27 S2: 8 |
| Wave 9 *Variables: 74* |  |  |  |  |  |  |  |  | S1: 39 S2: 5 |
| STAGE 3 without extra variables* | S3: 5 (out of 15 in total from S2) | S3: 7 (out of 22 in total from S2) | S3: 11 (out of 37 in total from S2) | S3: 10 (out of 46 in total from S2) | S3: 9 (out of 49 in total from S2) | S3: 12 (out of 65 in total from S2) | S3: 12 (out of 79 in total from S2) | S3: 9 (out of 82 in total from S2) | S3: 13 (out of 90 in total from S2) |
| Total with extra variables† | 7 | 9 | 14 | 13 | 12 | 15 | 15 | 14 | 16 |

***Table S2****: Number of variables selected in Stage 1 (S1), Stage 2 (S2) and Stage 3 (S3) for predictors of non-response (NR) at each sweep from the previous sweeps*

*We do not include in these counts the variables sex, country of birth, participation in all previous sweeps and father’s socioeconomic status which were used directly in Stage 3

†We include in these counts the variables sex, country of birth, participation in all previous sweeps and father’s socioeconomic status

## ***Table S3****. Estimated risk ratios and 95% confidence intervals for predictors of non-response at sweep 2 (age 5) (n = 17468).*

| Sweep | Variable | RR | 95% CI |
| --- | --- | --- | --- |
| Sweep 1 | Marital Status |  |  |
| (age 0) | Single | 1.00 | (reference) |
|  | Married | 0.55 | 0.51, 060 |
|  | Divorced/Separated | 0.78 | 0.66, 0.92 |
|  | Parity |  |  |
|  | 0 | 1.00 | (reference) |
|  | 1 | 1.03 | 0.96, 1.10 |
|  | 2 | 1.12 | 1.03, 1.22 |
|  | 3 | 1.17 | 1.05, 1.30 |
|  | >4 | 1.28 | 1.15, 1.42 |
|  | Father’s social status |  |  |
|  | I Single, no work, unskilled other | 1.00 | (reference) |
|  | II partial work | 0.87 | 0.78, 0.97 |
|  | III manual work | 0.83 | 0.75, 0.92 |
|  | III non-manual work | 0.86 | 0.77, 0.97 |
|  | IV managerial/technical work | 0.93 | 0.83, 1.06 |
|  | V professorial work | 0.81 | 0.69, 0.95 |
|  | Country of Birth |  |  |
|  | England | 1.00 | (reference) |
|  | Wales | 0.69 | 0.59, 0.81 |
|  | Scotland | 1.13 | 1.04, 1.23 |
|  | Other | 3.09 | 2.89, 3.31 |
|  | Father’s age at completion of education |  |  |
|  | ≤15 year old | 1.00 | (reference) |
|  | 16-18 year old | 1.13 | 1.06, 1.22 |
|  | ≥19 years old | 1.45 | 1.30, 1.62 |
|  | Number of antenatal visits |  |  |
|  | Continuous (per visit) | 0.98 | 0.97, 0.98 |
|  | Method of contraception |  |  |
|  | None | 1.00 | (reference) |
|  | Pill alone | 0.91 | 0.84, 0.98 |
|  | Pill alone and other method | 0.90 | 0.75, 1.08 |
|  | Other method | 0.80 | 0.74, 0.87 |

## ***Table S4****. Estimated risk ratios and 95% confidence intervals for predictors of non-response at sweep 3 (age 10) (n = 17447).*

| Sweep | Variable | RR | 95% CI |
| --- | --- | --- | --- |
| Sweep 1 | Marital Status |  |  |
| (age 0) | Single | 1.00 | (reference) |
|  | Married | 0.61 | 0.54, 0.68 |
|  | Divorced/Separated | 0.88 | 0.71, 1.10 |
|  | Where was mother-date of birth |  |  |
|  | Consultant bed/ GP bed | 1.00 | (reference) |
|  | NHS unit | 0.81 | 0.72, 0.91 |
|  | Own home | 0.97 | 0.59, 1.59 |
|  | Other | 1.19 | 0.94, 1.09 |
|  | Country of Birth |  |  |
|  | England | 1.00 | (reference) |
|  | Wales | 0.65 | 0.53, 0.80 |
|  | Scotland | 0.71 | 0.63, 0.81 |
|  | Other | 0.63 | 0.53, 0.75 |
|  | Father’s age at completion of education |  |  |
|  | ≤15 year old | 1.00 | (reference) |
|  | 16-18 year old | 1.24 | 1.13, 1.35 |
|  | ≥19 years old | 1.44 | 1.27, 1.64 |
| Sweep 2 | Ethnic group (mother)  Non-european vs european | 1.55 | 1.31, 1.82 |
| (age 7) | Attitude to childhood independence  Per Unit increase in z-score | 0.92 | 0.88, 0.97 |
|  | Tenure of accommodation |  |  |
|  | Owned | 1.00 | (reference) |
|  | Bought | 0.85 | 0.73, 0.98 |
|  | Council rented | 0.79 | 0.68, 0.93 |
|  | Other | 1.07 | 0.91, 1.25 |
|  | Household moves (per move) | 1.10 | 1.06, 1.14 |
| Non response at  Previous sweeps  (i.e. sweep 2) |  | 4.53 | 4.20, 4.89 |

Results from sequential multiple imputation analyses in which potential predictors of non-response at a given sweep are adjusted for previously identified potential predictors of non-response at that sweep and previous sweeps (i.e. not at subsequent sweeps).

## ***Table S5****. Estimated risk ratios and 95% confidence intervals for predictors of non-response at sweep 4 (age 16) (n = 17414).*

| Sweep | Variable | RR | 95% CI |
| --- | --- | --- | --- |
| Sweep 1 | Father’s social status |  |  |
| (age 0) | I Single, no work, unskilled other | 1.00 | (reference) |
|  | II partial work | 0.96 | 0.88, 1.04 |
|  | III manual work | 0.94 | 0.87, 1.01 |
|  | III non-manual work | 0.86 | 0.78, 0.94 |
|  | IV managerial/technical work | 0.87 | 0.79, 0.95 |
|  | V professorial work | 0.82 | 0.73, 0.93 |
|  | Country of Birth |  |  |
|  | England | 1.00 | (reference) |
|  | Wales | 0.70 | 0.62, 0.79 |
|  | Scotland | 0.89 | 0.83, 0.96 |
|  | Other | 0.65 | 0.57, 0.74 |
|  | Age of mother at first birth |  |  |
|  | Per year | 0.99 | 0.98, 0.99 |
|  | Method of contraception |  |  |
|  | None | 1.00 | (reference) |
|  | Pill alone | 0.91 | 0.86, 0.97 |
|  | Pill & Other method | 0.95 | 0.83, 1.10 |
|  | Other Method | 0.88 | 0.83, 0.94 |
|  | Parity |  |  |
|  | 0 | 1.00 | (reference) |
|  | 1 | 1.09 | 1.03, 1.15 |
|  | 2 | 1.10 | 1.03, 1.17 |
|  | 3 | 1.12 | 1.03, 1.22 |
|  | >4 | 1.19 | 1.10, 1.28 |
|  | Certainty of last menstrual period |  |  |
|  | Certain vs uncertain | 1.10 | 1.05, 1.16 |
|  | Where was mother-date of birth |  |  |
|  | Consultant bed/ GP bed | 1.00 | (reference) |
|  | NHS unit | 0.84 | 0.79, 0.90 |
|  | Own home | 0.83 | 0.62, 1.11 |
|  | Other | 1.16 | 1.02, 1.32 |
| Sweep 2  (Age 6) | Type of accommodation |  |  |
|  | Detached | 1.00 | (reference) |
|  | Semi-detached | 0.95 | 0.88, 1.04 |
|  | Terrace | 1.02 | 0.93, 1.11 |
|  | Flat-Maisonette | 1.18 | 1.07, 1.30 |
|  | Rooms-other | 1.02 | 0.87, 1.20 |
|  |  |  |  |
|  | Ethnic group (father) |  |  |
|  | European UK | 1.00 | (reference) |
|  | European EU | 1.28 | 1.13, 1.45 |
|  | Other | 1.26 | 1.16, 1.37 |
|  | Neighbourhood group |  |  |
|  | Poor | 1.00 | (reference) |
|  | Average | 0.88 | 0.82, 0.95 |
|  | Well to do | 0.84 | 0.76, 0.93 |
|  | Rural | 0.78 | 0.70, 0.86 |
| Sweep 3  (Age 10) | Accommodation occupied by family |  |  |
|  | Flat self-contained | 1.00 | (reference) |
|  | House | 0.70 | 0.62, 0.79 |
|  | Other | 0.74 | 0.57, 0.95 |
|  | Estimated reading age (in years) |  |  |
|  | Continuous (per year) | 0.94 | 0.91, 0.96 |
|  | BAS Matrix - Total score |  |  |
|  | Continuous (per unit) | 0.98 | 0.98, 0.99 |
| Non response at  Previous sweeps  (i.e. sweeps 2 & 3) |  | 1.54 | 1.43, 1.66 |

Results from sequential multiple imputation analyses in which potential predictors of non-response at a given sweep are adjusted for previously identified potential predictors of non-response at that sweep and previous sweeps (i.e. not at subsequent sweeps).

## ***Table S6****. Estimated risk ratios and 95% confidence intervals for predictors of non-response at sweep 5 (age 26) (n = 17287).*

| Sweep | Variable | RR | 95% CI |
| --- | --- | --- | --- |
| Sweep 1 | Father’s social status |  |  |
| (age 0) | I Single, no work, unskilled other | 1.00 | (reference) |
|  | II partial work | 0.95 | 0.90, 1.00 |
|  | III manual work | 0.90 | 0.85, 0.94 |
|  | III non-manual work | 0.83 | 0.78, 0.89 |
|  | IV managerial/technical work | 0.82 | 0.76, 0.88 |
|  | V professorial work | 0.83 | 0.75, 0.91 |
|  | Age of mother at 1^st^ birth |  |  |
|  | Continuous (per year) | 0.99 | 0.98, 0.99 |
|  | Method of contraception |  |  |
|  | None | 1.00 | (reference) |
|  | Pill alone | 0.92 | 0.88, 0.96 |
|  | Pill & Other method | 0.91 | 0.82, 1.02 |
|  | Other Method | 0.88 | 0.84, 0.91 |
|  | Parity (i.e. number of older siblings) |  |  |
|  | 0 | 1.00 | (reference) |
|  | 1 | 1.00 | 0.96, 1.05 |
|  | 2 | 1.04 | 0.99, 1.10 |
|  | 3 | 1.12 | 1.05, 1.19 |
|  | >4 | 1.16 | 1.10, 1.23 |
|  | Certainty of last menstrual period |  |  |
|  | Certain vs uncertain | 1.07 | 1.03, 1.12 |
|  | Sex |  |  |
|  | Female vs male | 0.78 | 0.75, 0.80 |
| Sweep 2  (Age 6) | External score |  |  |
|  |  | 1.03 | 1.02, 1.04 |
|  | Harris scoring method |  |  |
|  | Per unit increase | 0.98 | 0.98, 0.99 |
| Sweep 3  (Age 10) | Gross family income | 0.97 | 0.96, 0.99 |
|  | Teacher Rutter assessment | 1.02 | 1.01, 1.03 |
|  | Number of household accessories | 0.98 | 0.96, 0.99 |
| Sweep 4  (Age 16) | Satisfaction with teen’s school progress |  |  |
|  | Very satisfied | 1.00 | (reference) |
|  | Fairly satisfied | 1.10 | 1.04, 1.16 |
|  | Not satisfied | 1.20 | 1.11, 1.29 |
| Non response at  Previous sweeps  (i.e. sweeps 2-4) |  | 1.55 | 1.50, 1.61 |

Results from sequential multiple imputation analyses in which potential predictors of non-response at a given sweep are adjusted for previously identified potential predictors of non-response at that sweep and previous sweeps (i.e. not at subsequent sweeps).

## ***Table S7****. Estimated risk ratios and 95% confidence intervals for predictors of non-response at sweep 6 (age 30) (n = 17035).*

| Sweep | Variable | RR | 95% CI |
| --- | --- | --- | --- |
| Sweep 1 | Father’s social status |  |  |
| (age 0) | I Single, no work, unskilled other | 1.00 | (reference) |
|  | II partial work | 0.93 | 0.86, 1.01 |
|  | III manual work | 0.86 | 0.80, 0.92 |
|  | III non-manual work | 0.87 | 0.79, 0.95 |
|  | IV managerial/technical work | 0.84 | 0.77, 0.92 |
|  | V professorial work | 0.85 | 0.75, 0.96 |
|  | Sex |  |  |
|  | Female vs male | 0.86 | 0.83, 0.90 |
|  | Number of antenatal Visits |  |  |
|  | Per visit | 0.99 | 0.98, 0.99 |
|  | Parity (i.e. number of older siblings) |  |  |
|  | 0 | 1.00 | (reference) |
|  | 1 | 0.93 | 0.88, 0.98 |
|  | 2 | 0.96 | 0.90, 1.03 |
|  | 3 | 0.97 | 0.89, 1.05 |
|  | >4 | 1.12 | 1.04, 1.21 |
| Sweep 2  (Age 6) | Ethnic group (mother)  Non-european vs european | 1.18 | 1.07, 1.29 |
|  | Household moves |  |  |
|  | Per move | 1.04 | 1.02, 1.06 |
|  | Neighbourhood group |  |  |
|  | Poor | 1.00 | (reference) |
|  | Average | 0.93 | 0.87, 1.02 |
|  | Well to do | 0.81 | 0.73, 0.89 |
|  | Rural | 0.80 | 0.71, 0.89 |
| Sweep 3  (Age 10) | Score BAS Matrices | 0.99 | 0.98, 0.99 |
| Sweep 4  (Age 26) | How easy would you quit a job, if there  was no other job to go to (range: 1-5) |  |  |
|  | Continuous (per unit) | 0.94 | 0.91, 0.97 |
|  | N of accidents elsewhere |  |  |
|  | 0 | 1.00 | (reference) |
|  | 1 | 1.01 | 0.91, 1.12 |
|  | ≥2 | 1.34 | 1.14, 1.57 |
|  | Tenure of current address |  |  |
|  | Own/ Parents (rent-free) | 1.00 | (reference) |
|  | Buying on morgage | 0.75 | 0.65, 0.87 |
|  | Rent | 1.03 | 0.92, 1.17 |
|  | Other | 1.04 | 0.88, 1.24 |
| Non response at  Previous sweeps  (i.e. sweeps 2-5) |  | 3.58 | 3.31, 3.87 |

Results from sequential multiple imputation analyses in which potential predictors of non-response at a given sweep are adjusted for previously identified potential predictors of non-response at that sweep and previous sweeps (i.e. not at subsequent sweeps).

## ***Table S8****. Estimated risk ratios and 95% confidence intervals for predictors of non-response at sweep 7 (age 30) (n = 16785).*

| Sweep | Variable | RR | 95% CI |
| --- | --- | --- | --- |
| Sweep 1 | Father’s social status |  |  |
| (age 0) | I Single, no work, unskilled other | 1.00 | (reference) |
|  | II partial work | 0.95 | 0.90, 1.02 |
|  | III manual work | 0.92 | 0.87, 0.98 |
|  | III non-manual work | 0.88 | 0.82, 0.95 |
|  | IV managerial/technical work | 0.86 | 0.79, 0.92 |
|  | V professorial work | 0.87 | 0.78, 0.96 |
|  | Sex |  |  |
|  | Female vs male | 0.90 | 0.87, 0.93 |
|  | Ever a teenager mother |  |  |
|  | 0-19 | 1.00 | (reference) |
|  | 20 plus | 0.91 | 0.88, 0.95 |
|  | Parity (i.e. number of older siblings) |  |  |
|  | 0 | 1.00 | (reference) |
|  | 1 | 1.02 | 0.98, 1.07 |
|  | 2 | 1.09 | 1.04, 1.15 |
|  | 3 | 1.10 | 1.03, 1.18 |
|  | >4 | 1.18 | 1.11, 1.26 |
|  | Present marital status |  |  |
|  | Single | 1.00 | (reference) |
|  | Married | 0.81 | 0.76, 0.85 |
|  | Widowed/Divorced/Separated | 0.90 | 0.80, 1.00 |
|  | Method of contraception |  |  |
|  | None | 1.00 | (reference) |
|  | Pill alone | 0.94 | 0.90, 0.99 |
|  | Pill & Other method | 0.89 | 0.79, 1.01 |
|  | Other Method | 0.86 | 0.82, 0.90 |
| Sweep 2  (Age 6) | Household moves  Per move | 1.04 | 1.02, 1.05 |
|  | Number of household accessories |  |  |
|  | Per accessory | 0.96 | 0.95, 0.98 |
| Sweep 3  (Age 10) | Maths Test Score  Per mark | 0.99 | 0.99, 0.99 |
| Sweep 4  (Age 16) | Maths 0 level or equivalent  No vs yes | 1.17 | 1.07, 1.27 |
| Sweep 5  (Age 26) | Satisfaction about life (1-3) |  |  |
|  | per unit increase | 0.94 | 0.90, 0.97 |
| Sweep 6  (Age 30) | Does participant intend to move?  No vs Yes | 0.86 | 0.82, 0.90 |
|  | Did you vote in the 97 elections?  No vs Yes | 1.14 | 1.08, 1.20 |
|  | Do you like the area? (Units 1-5)  Per unit | 1.04 | 1.02, 1.06 |
| Non response at  Previous sweeps  (i.e. sweeps 2-6) |  | 3.54 | 3.29, 3.81 |

## ***Table S9****. Estimated risk ratios and 95% confidence intervals for predictors of non-response at sweep 8 (age 34) (n = 16699).*

| Sweep | Variable | RR | 95% CI |
| --- | --- | --- | --- |
| Sweep 1 | Father’s social status |  |  |
| (age 0) | I Single, no work, unskilled other | 1.00 | (reference) |
|  | II partial work | 0.97 | 0.92, 1.03 |
|  | III manual work | 0.94 | 0.89, 0.99 |
|  | III non-manual work | 0.89 | 0.83, 0.95 |
|  | IV managerial/technical work | 0.84 | 0.78, 0.90 |
|  | V professorial work | 0.80 | 0.72, 0.88 |
|  | Sex |  |  |
|  | Female vs male | 0.91 | 0.88, 0.94 |
|  | N of antenatal visits |  |  |
|  | Continuous (per visit) | 0.99 | 0.99, 1.00 |
|  | Parity (i.e. number of older siblings) |  |  |
|  | 0 | 1.00 | (reference) |
|  | 1 | 0.99 | 0.95, 1.03 |
|  | 2 | 1.03 | 0.99, 1.08 |
|  | 3 | 1.12 | 1.06, 1.19 |
|  | >4 | 1.16 | 1.10, 1.23 |
|  | Certainty of Data of last Menstrual Period |  |  |
|  | Certain vs uncertain | 1.12 | 1.08, 1.16 |
|  | Method of contraception |  |  |
|  | None | 1.00 | (reference) |
|  | Pill alone | 0.94 | 0.90, 0.98 |
|  | Pill & Other method | 0.90 | 0.81, 1.00 |
|  | Other method | 0.90 | 0.86, 0.94 |
| Sweep 2  (Age 6) | Household moves |  |  |
|  | Per move | 1.04 | 1.02, 1.05 |
|  | Copying design score |  |  |
|  | Per unit | 0.97 | 0.96, 0.98 |
| Sweep 3  (Age 10) | Maths Test Score  Continuous (Per mark) | 0.99 | 0.99, 1.00 |
|  | BASMATRIX  Continuous (Per unit) | 0.99 | 0.98, 0.99 |
| Sweep 5  (Age 26) | Age left education |  |  |
|  | 12-16 | 1.00 | (reference) |
|  | 17-18 | 0.88 | 0.80, 0.96 |
|  | 19 plus | 1.44 | 1.37, 1.52 |
| Sweep 6  (Age 30) | Does participant intend to have more children |  |  |
|  | Yes | 1.00 | (reference) |
|  | No | 1.13 | 1.08, 1.18 |
|  | Don’t know | 1.09 | 1.04, 1.15 |
|  | Financial situation |  |  |
|  | Living comfortably | 1.00 | (reference) |
|  | Doing alright | 0.97 | 0.92, 1.02 |
|  | Just about getting by | 1.00 | 0.95, 1.06 |
|  | Finding it quite difficult | 1.13 | 1.06, 1.21 |
|  | Finding it very difficult | 1.08 | 0.98, 1.19 |
|  | Voted in general elections 1997? |  |  |
|  | No vs yes | 1.13 | 1.08, 1.17 |
| Non response at  Previous sweeps  (i.e. sweeps 2-7) |  | 3.36 | 3.09, 3.64 |

Results from sequential multiple imputation analyses in which potential predictors of non-response at a given sweep are adjusted for previously identified potential predictors of non-response at that sweep and previous sweeps (i.e. not at subsequent sweeps).

## ***Table S10****. Estimated risk ratios and 95% confidence intervals for predictors of non-response at sweep 9 (age 42) (n = 16638).*

| Sweep | Variable | RR | 95% CI |
| --- | --- | --- | --- |
| Sweep 1 | Father’s social status |  |  |
| (age 0) | I Single, no work, unskilled other | 1.00 | (reference) |
|  | II partial work | 0.95 | 0.89, 1.01 |
|  | III manual work | 0.90 | 0.85, 0.96 |
|  | III non-manual work | 0.87 | 0.81, 0.94 |
|  | IV managerial/technical work | 0.79 | 0.73, 0.86 |
|  | V professorial work | 0.79 | 0.70, 0.88 |
|  | Country of Birth |  |  |
|  | England | 1.00 | (reference) |
|  | Wales | 1.01 | 0.94, 1.10 |
|  | Scotland | 1.14 | 1.08, 1.20 |
|  | Other | 0.98 | 0.88, 1.08 |
|  | Sex |  |  |
|  | Female vs male | 0.91 | 0.88, 0.94 |
|  | N of antenatal visits |  |  |
|  | Continuous (per visit) | 0.99 | 0.99, 1.00 |
|  | Certainty of Data of last Menstrual Period |  |  |
|  | Certain vs uncertain | 1.09 | 1.05, 1.14 |
| Sweep 2  (Age 6) | Copying design score  Continuous (Per unit) | 0.97 | 0.96, 0.98 |
| Sweep 3  (Age 10) | Score BAS Matrices  Per mark | 0.98 | 0.98, 0.99 |
| Sweep 6  (Age 30) | Voted in general elections 1997?  No vs Yes | 1.11 | 1.05, 1.17 |
| Sweep 7  (Age 34) | Work overtime?  No vs Yes | 1.17 | 1.09, 1.25 |
|  | Should everyone behave responsibly?  No vs Yes | 1.26 | 1.12, 1.42 |
|  | How many times have you been found guilty in a criminal court?  ≥1 vs 0 | 0.56 | 0.46, 0.69 |
| Sweep 8  (Age 42) | Participant willing to be contacted for parents’ research project?  No vs yes | 1.30 | 1.19, 1.42 |
| Non response at  Previous sweeps  (i.e. sweeps 2-8) |  | 6.50 | 5.74, 7.35 |

Results from sequential multiple imputation analyses in which potential predictors of non-response at a given sweep are adjusted for previously identified potential predictors of non-response at that sweep and previous sweeps (i.e. not at subsequent sweeps).

## **Table S11**. Estimated risk ratios and 95% confidence intervals for predictors of non-response at sweep 10 (age 46) (n = 16585).

| Sweep | Variable | RR | 95% CI |
| --- | --- | --- | --- |
| Sweep 1 | Father’s social status |  |  |
| (age 0) | I Single, no work, unskilled other | 1.00 | (reference) |
|  | II partial work | 0.97 | 0.92, 1.03 |
|  | III manual work | 0.90 | 0.86, 0.95 |
|  | III non-manual work | 0.88 | 0.82, 0.94 |
|  | IV managerial/technical work | 0.79 | 0.74, 0.85 |
|  | V professorial work | 0.77 | 0.70, 0.85 |
|  | Country of Birth |  |  |
|  | England | 1.00 | (reference) |
|  | Wales | 1.04 | 0.98, 1.11 |
|  | Scotland | 1.12 | 1.08, 1.18 |
|  | Other | 0.93 | 0.85, 1.02 |
|  | Parity (i.e. number of older siblings) |  |  |
|  | 0 | 1.00 | (reference) |
|  | 1 | 1.02 | 0.98, 1.06 |
|  | 2 | 1.06 | 1.01, 1.11 |
|  | 3 | 1.11 | 1.05, 1.17 |
|  | >4 | 1.12 | 1.06, 1.18 |
|  | Certainty of last menstrual period |  |  |
|  | Certain vs uncertain | 1.08 | 1.04, 1.12 |
|  | Was lactation attempted |  |  |
|  | Not attempted vs attempted | 1.06 | 1.03, 1.10 |
|  | Number of antenatal visits |  |  |
|  | Per visit | 0.99 | 0.99, 1.00 |
| Sweep 2  (Age 6) | Copying designs score  Continuous (Per unit) | 0.97 | 0.96, 0.99 |
| Sweep 3  (Age 10) | Score BAS Matrices  Continuous (Per unit) | 0.98 | 0.98, 0.99 |
|  | Accommodation |  |  |
|  | Owned | 1.00 | (reference) |
|  | Bought | 0.95 | 0.89, 1.00 |
|  | Council rented | 1.04 | 0.98, 1.10 |
|  | Other rented | 1.01 | 0.92, 1.12 |
|  | Tied to occupation | 0.95 | 0.84, 1.07 |
| Sweep 6  (Age 30) | Had eczema or skin problems?  No vs yes | 1.10 | 1.04, 1.15 |
|  | Voted in general elections 1997? |  |  |
|  | No vs yes | 1.10 | 1.06, 1.14 |
| Sweep 7  (Age 34) | Is this address participant’s residence?  No vs yes | 1.11 | 1.05, 1.18 |
| Sweep 8  (Age 38) | Willing to be contacted for Parents Research Project  No vs Yes | 1.21 | 1.15, 1.28 |
|  | Any children aged 0-6 |  |  |
|  | No vs Yes | 1.11 | 1.05, 1.18 |
| Sweep 9  (Age 42) | Total score |  |  |
|  | Per mark | 0.98 | 0.97, 0.99 |
| Non response at  Previous sweeps  (i.e. sweeps 2-9) |  | 4.55 | 4.14, 5.00 |

Results from sequential multiple imputation analyses in which potential predictors of non-response at a given sweep are adjusted for previously identified potential predictors of non-response at that sweep and previous sweeps (i.e. not at subsequent sweeps).

**Section 2: Further details on important predictors of non-response for every sweep. Variable description and information about how each important predictor of non-response was modelled in the imputation stage (3rd stage of identifying important predictors of non-response)**

**Table S12**. Predictors of non-response at Sweep 2 (age 5).

| **Sweep** | **Variable description** | **Variable** | **Variable derivation details** | **How variable was modelled in the imputation stage (3^rd^ stage of identifying important predictors of non-response)** |
| --- | --- | --- | --- | --- |
| Sweep 1 (age 0) | Marital Status | a0012 | Single  Married  Divorced/Separated | Multinomial logistic regression |
|  | Parity | A0166_new | Recoded from A0166    0  1  2  3  ≥4 | Multinomial logistic regression |
|  | Father’s social status | BD1BPOS_new | Recoded from BD1BPOS    I Single, no work, unskilled other  II partial work  III manual work  III non-manual work  IV managerial/technical work  V professorial work | Multinomial logistic regression |
|  | Country of Birth | COB_new | Recoded from COB    England  Wales  Scotland  Other | N/A (no missing data) |
|  | Father’s age at completion of education | A0010_new | Recoded from a0010    ≤15 year old  16-18 year old  ≥19 years old | Multinomial logistic regression |
|  | Number of antenatal visits | a0190 | Continuous (per visit) | Negative binomial |
|  | Method of contraception | a0029b | None  Pill alone  Pill alone and other method  Other method | Multinomial logistic regression |

**Table S13**. Predictors of non-response at Sweep 3 (age 10).

| **Sweep** | **Variable description** | **Variable** | **Variable derivation details** | **How variable was modelled in the imputation stage (3^rd^ stage of identifying important predictors of non-response)** |
| --- | --- | --- | --- | --- |
| Sweep 1 (age 0) | Marital Status | a0012 | Single  Married  Divorced/Separated | Multinomial logistic regression |
|  | Where was mother-date of birth | A0357_new | Recoded from A0357    Consultant bed/ GP bed  NHS unit  Own home  Other | Multinomial logistic regression |
|  | Country of Birth |  | England  Wales  Scotland  Other | N/A (no missing data) |
|  | Father’s age at completion of education | A0010_new | Recoded from a0010    ≤15 year old  16-18 year old  ≥19 years old | Multinomial logistic regression |
| Sweep 2 (age 5) | Ethnic group (mother) | E246a_new | Recoded from e246a    Non-european vs european | Logistic regression |
|  | Attitude to childhood independence | d124h | Per Unit increase in z-score | Linear regression |
|  | Tenure of accommodation | E220_new | Recoded from e220    Owned  Bought  Council rented  Other | Multinomial logistic regression |
|  | Household moves (ordered) | E249_new | Recoded from e249    0  1  2  3  4  >5 | Ordinal logistic regression |
| Non-response at  Previous sweeps  (i.e. sweep 2) |  |  | Yes vs no | Logistic regression |

**Table S14**. Predictors of non-response at Sweep 4 (age 16).

| **Sweep** | **Variable description** | **Variable** | **Variable derivation details** | **How variable was modelled in the imputation stage (3^rd^ stage of identifying important predictors of non-response)** |
| --- | --- | --- | --- | --- |
| Sweep 1 (age 0) | Father’s social status | BD1PSOC_new | Recoded from BD1PSOC    I Single, no work, unskilled other  II partial work  III manual work  III non-manual work  IV managerial/technical work  V professorial work | Multinomial logistic regression |
|  | Country of Birth | COB_new | Recoded from COB    England  Wales  Scotland  Other | N/A (no missing data) |
|  | Age of mother at first birth | BD1AGEFB | Per year | Truncated linear regression  Lower Limit 12,  Upper limit: 47 |
|  | Method of contraception | a0029b | None  Pill alone  Pill & Other method  Other Method | Multinomial logistic regression |
|  | Parity | A0166_new | Recoded from A0166  0  1  2  3  >4 | Multinomial logistic regression |
|  | Certainty of last menstrual period | a0196 | Certain vs uncertain | Multinomial logistic regression |
|  | Where was mother-date of birth | A357_new | Recoded from A0357    Consultant bed/ GP bed  NHS unit  Own home  Other | Multinomial logistic regression |
| Sweep 2 (age 5) | Type of accommodation | E218_new | Recoded from E218    Detached  Semi-detached  Terrace  Flat-Maisonette  Rooms-other | Multinomial logistic regression |
|  | Ethnic group (father) | E246b_new | Recoded from E246b  European UK  European EU  Other | Multinomial logistic regression |
|  | Neighbourhood group | e267b | Poor  Average  Well to do  Rural | Multinomial logistic regression |
| Sweep 3 (age 10) | Accommodation occupied by family | D1_1_new | Recoded from D1_1l    Flat self-contained  House  Other | Multinomial logistic regression |
|  | Estimated reading age (in years) | bd3rdage | Continuous (per year) | Linear regression |
|  | British Ability Scales Matrix - Total score | BASmatrx | Continuous (per unit) |  |
| Non-response at  Previous sweeps  (i.e. sweeps 2 & 3) |  |  | Yes vs no | Logistic regression |

**Table S15**. Predictors of non-response at Sweep 5 (age 26).

| **Sweep** | **Variable description** | **Variable** | **Variable derivation details** | **How variable was modelled in the imputation stage (3^rd^ stage of identifying important predictors of non-response)** |
| --- | --- | --- | --- | --- |
| Sweep 1 (age 0) | Father’s social status | BD1PSOC_new | Recoded from BD1PSOC    I Single, no work, unskilled other  II partial work  III manual work  III non-manual work  IV managerial/technical work  V professorial work | Multinomial logistic regression |
|  | Age of mother at 1^st^ birth | BD1AGEFB | Continuous (per year) | Truncated linear regression  Lower Limit 12,  Upper limit: 47 |
|  | Method of contraception | a0029b | None  Pill alone  Pill & Other method  Other Method | Multinomial logistic regression |
|  | Parity (i.e. number of older siblings) | A0166_new | Recoded from a0166    0  1  2  3  >4 | Multinomial logistic regression |
|  | Certainty of last menstrual period | a0196 | Certain vs uncertain | Multinomial logistic regression |
|  | Sex | SEX | Female vs male | N/A (no missing data) |
| Sweep 2 (age 5) | External score | Extern_score_5 | Per unit increase | Ordinal logistic regression |
|  | Harris scoring method | f114 | Per unit increase | Truncated linear regression  Lower Limit 1,  Upper limit: 23 |
| Sweep 3 (age 10) | Gross family income | grfaminc | Per unit increase | Ordinal logistic regression |
|  | Teacher Rutter assessment | B3T_Rutt | Per unit increase | Negative Binomial regression |
|  | Number of household accessories | b3hldstf | Per unit increase | Truncated linear regression  Lower Limit 0,  Upper limit: 10 |
| Sweep 4 (age 16) | Satisfaction with teen’s school progress | Pb3_1 | Very satisfied  Fairly satisfied  Not satisfied | Multinomial logistic regression |
| Non-response at  Previous sweeps  (i.e. sweeps 2, 3 & 4) |  |  | Yes vs no | Logistic regression |

**Table S16**. Predictors of non-response at Sweep 6 (age 29).

| **Sweep** | **Variable description** | **Variable** | **Variable derivation details** | **How variable was modelled in the imputation stage (3^rd^ stage of identifying important predictors of non-response)** |
| --- | --- | --- | --- | --- |
| Sweep 1 (age 0) | Father’s social status | BD1PSOC_new | Recoded from BD1PSOC    I Single, no work, unskilled other  II partial work  III manual work  III non-manual work  IV managerial/technical work  V professorial work | Multinomial logistic regression |
|  | Sex | SEX | Female vs male | N/A (no missing data) |
|  | Number of antenatal Visits | a0190 | Per visit | Negative Binomial regression |
|  | Parity (i.e. number of older siblings) | A0166_new | Recoded from A0166    0  1  2  3   >4 | Multinomial logistic regression |
| Sweep 2 (age 5) | Ethnic group (mother) | E246a_new | Recoded from E246a    Non-european vs european | Multinomial logistic regression |
|  | Household moves | E249_new | Recoded from e249    0  1  2  3  4  >5 | Ordinal logistic regression |
|  | Neighbourhood group | e267b | Poor  Average  Well to do  Rural | Multinomial logistic regression |
| Sweep 3 (age 10) | Score BAS Matrices | BASmatrx | Per move | Linear regression |
| Sweep 4 (age 16) |  |  |  |  |
| Sweep 5 (age 26) | How easy would you quit a job, if there  was no other job to go to (range: 1-5) | B960421_new | Continuous (per unit) | Ordinal logistic regression |
|  | N of accidents elsewhere | Aelse_new | Recoded from aelse    0  1  ≥2 | Multinomial logistic regression |
|  | Tenure of current address | B960421_new | Recoded from B960421    Own/ Parents (rent-free)  Buying on morgage  Rent  Other | Multinomial logistic regression |
| Non-response at  Previous sweeps  (i.e. sweeps 2-5) |  |  | Yes vs no | Logistic regression |

**Table S17**. Predictors of non-response at Sweep 7 (age 34).

| **Sweep** | **Variable description** | **Variable** | **Variable derivation details** | **How variable was modelled in the imputation stage (3^rd^ stage of identifying important predictors of non-response)** |
| --- | --- | --- | --- | --- |
| Sweep 1 (age 0) | Father’s social status | BD1PSOC_new | Recoded from BD1PSOC    I Single, no work, unskilled other  II partial work  III manual work  III non-manual work  IV managerial/technical work  V professorial work | Multinomial logistic regression |
|  | Sex | SEX | Female vs male | N/A (no missing data) |
|  | Ever a teenager mother | BD1_TEENM | 0-19  20 plus | Multinomial logistic regression |
|  | Parity (i.e. number of older siblings) | A0166_new | Recoded from a0166    0  1  2  3  >4 | Multinomial logistic regression |
|  | Present marital status | a0012 | Single  Married  Widowed/Divorced/Separated | Multinomial logistic regression |
|  | Method of contraception | a0029b | None  Pill alone  Pill & Other method  Other Method | Multinomial logistic regression |
| Sweep 2 (age 5) | Household moves | E249_new | Recoded from e249    0  1  2  3  4  >5 | Ordinal logistic regression |
|  | Number of household accessories | hhldstuf | Per accessory | Truncated linear regression  Lower Limit 0,  Upper limit: 8 |
| Sweep 3 (age 10) | Maths Test Score | BD3MATHS | Per mark | Linear regression |
| Sweep 4 (age 16) | Maths 0 level or equivalent | T2c1_1 | No vs yes | Multinomial logistic regression |
| Sweep 5 (age 26) | Satisfaction about life (1-3) ordered | B960666_new | Recoded from B960666    1-->0-5  2-->6-7  3-->8-9 | Ordinal logistic regression |
| Sweep 6 (age 29) | Does participant intend to move? | Wantmove_new | Recoded from Wantmove (8,9 turned to missing)    No vs yes | Multinomial logistic regression |
|  | Did you vote in the 97 elections? | Vote97_new | Recoded from vote97 (8,9 turned to missing)  No vs yes | Multinomial logistic regression |
|  | Do you like the area? (Units 1-5) | Likearea_new | Recoded from likearea (8,9 turned to missing)  No vs yes | Ordinal logistic regression |
| Non-response at  Previous sweeps  (i.e. sweeps 2-6) |  |  | Yes vs no | Logistic regression |

**Table S18**. Predictors of non-response at Sweep 8 (age 38).

| **Sweep** | **Variable description** | **Variable** | **Variable derivation details** | **How variable was modelled in the imputation stage (3^rd^ stage of identifying important predictors of non-response)** |
| --- | --- | --- | --- | --- |
| Sweep 1 (age 0) | Father’s social status | BP1PSOC_new | Recoded from BP1PSOC    I Single, no work, unskilled other  II partial work  III manual work  III non-manual work  IV managerial/technical work  V professorial work | Multinomial logistic regression |
|  | Sex | SEX | Female vs male | N/A (no missing data) |
|  | N of antenatal visits | a0190 | Continuous (per visit) | Negative binomial |
|  | Parity (i.e. number of older siblings) | A0166_new | Recoded from a0166    0  1  2  3  >4 | Multinomial logistic regression |
|  | Certainty of Data of last Menstrual Period | a0196 | Certain vs uncertain | Multinomial logistic regression |
|  | Method of contraception | a0029b | None  Pill alone  Pill & Other method  Other method | Multinomial logistic regression |
| Sweep 2 (age 5) | Household moves | E249_new | Recoded from e249    0  1  2  3  4  >5 | Ordinal logistic regression |
|  | Copying design score | f119 | Per unit | Truncated linear regression  Lower Limit 0,  Upper limit: 8 |
| Sweep 3 (age 10) | Maths Test Score | BD3MATHS | Per unit increase | Linear regression |
|  | Score BAS Matrices | BASMATRIX | Per unit increase | Linear regression |
| Sweep 4 (age 16) |  |  |  |  |
| Sweep 5 (age 26) | Age left education | B960132_new | Recoded from B960132    12-16  17-18  19 plus | Multinomial logistic regression |
| Sweep 6 (age 29) | Does participant intend to have more children | infertlc_new | Recoded from infertlc (9-->missing)    Yes  No  Don’t know | Multinomial logistic regression |
|  | Financial situation | Finnow_new | Recoded from finnow (8, 9 -->missing)    Living comfortably  Doing alright  Just about getting by  Finding it quite difficult  Finding it very difficult | Multinomial logistic regression |
|  | Voted in general elections 1997? | Vote97_new | Recoded from vote97 (8, 9 -->missing)    No vs yes | Multinomial logistic regression |
| Sweep 7 (age 34) |  |  |  |  |
| Non-response at  Previous sweeps  (i.e. sweeps 2-7) |  |  | Yes vs no | Logistic regression |

**Table S19**. Predictors of non-response at Sweep 9 (age 42).

| **Sweep** | **Variable description** | **Variable** | **Variable derivation details** | **How variable was modelled in the imputation stage (3^rd^ stage of identifying important predictors of non-response)** |
| --- | --- | --- | --- | --- |
| Sweep 1 (age 0) | Father’s social status | BD1PSOC_new | Recoded from BD1PSOC    I Single, no work, unskilled other  II partial work  III manual work  III non-manual work  IV managerial/technical work  V professorial work | Multinomial logistic regression |
|  | Country of Birth | COB_new | Recoded from COB    England  Wales  Scotland  Other | N/A (no missing data) |
|  | Sex | SEX | Female vs male | N/A (no missing data) |
|  | N of antenatal visits | a0190 | Continuous (per visit) | Negative binomial regression |
|  | Certainty of Data of last Menstrual Period | a0196 | Certain vs uncertain | Multinomial logistic regression |
| Sweep 2 (age 5) | Copying design score | f119 | Continuous (Per unit) | Truncated linear regression  Lower Limit 0,  Upper limit: 8 |
| Sweep 3 (age 10) | Score BAS Matrices | BASmatrx | Per mark | Linear regression |
| Sweep 4 (age 16) |  |  |  |  |
| Sweep 5 (age 26) |  |  |  |  |
| Sweep 6 (age 29) | Voted in general elections 1997? | Vote97_new | Recoded from vote97 (8, 9 -->missing)    No vs yes | Multinomial logistic regression |
| Sweep 7 (age 34) | Work overtime? | b7otimny | No vs Yes | Multinomial logistic regression |
|  | Should everyone behave responsibly? | B7_eresp6 | No vs Yes | Multinomial logistic regression |
|  | How many times have you been found guilty in a criminal court? | B7court_new | Recoded from B7court (8, 9 -->missing)    ≥1  vs 0 | Logistic regression |
| Sweep 8 (age 38) | Participant willing to be contacted for parents’ research project? | b8parent | No vs yes | Multinomial logistic regression |
| Non-response at  Previous sweeps  (i.e. sweeps 2-8) |  |  | Yes vs no | Logistic regression |

**Table S20**. Predictors of non-response at Sweep 10 (age 46).

| **Sweep** | **Variable description** | **Variable** | **Variable derivation details** | **How variable was modelled in the imputation stage (3^rd^ stage of identifying important predictors of non-response)** |
| --- | --- | --- | --- | --- |
| Sweep 1 (age 0) | Father’s social status | BD1PSOC_new | Recoded from BD1PSOC    I Single, no work, unskilled other  II partial work  III manual work  III non-manual work  IV managerial/technical work  V professorial work | Multinomial logistic regression |
|  | Country of Birth | COB_new | Recoded from COB    England  Wales  Scotland  Other | Multinomial logistic regression |
|  | Parity (i.e. number of older siblings) | A0166_new | Recoded from a0166    0  1  2  3  >4 | Multinomial logistic regression |
|  | Certainty of last menstrual period | a0196 | Certain vs uncertain | Multinomial logistic regression |
|  | Was lactation attempted | a0297 | Not attempted vs attempted | Multinomial logistic regression |
|  | Number of antenatal visits | a0190 | Per visit | Negative binomial regression |
| Sweep 2 (age 5) | Copying designs score | f119 | Continuous (Per unit) | Truncated linear regression  Lower Limit 0,  Upper limit: 8 |
| Sweep 3 (age 10) | Score BAS Matrices | BASmatrx | Continuous (Per unit) | Linear regression |
|  | Accommodation | D2_new | Recoded from d2    Owned  Bought  Council rented  Other rented  Tied to occupation | Multinomial logistic regression |
| Sweep 4 (age 16) |  |  |  |  |
| Sweep 5 (age 26) |  |  |  |  |
| Sweep 6 (age 29) | Had eczema or skin problems? | Othskin_new | Recoded from othskin (8,9-->missing)    No vs yes | Multinomial logistic regression |
|  | Voted in general elections 1997? | Vote97_new | Recoded from vote97 (8, 9 -->missing)    No vs yes | Multinomial logistic regression |
| Sweep 7 (age 34) | Is this address participant’s residence? | b7nrmal | No vs yes | Multinomial logistic regression |
| Sweep 8 (age 38) | Willing to be contacted for Parents Research Project | b8parent | No vs Yes | Multinomial logistic regression |
|  | Any children aged 0-6 | b8chd006 | No vs Yes | Multinomial logistic regression |
| Sweep 9 (age 42) | Total score | B9VSCORE | Per mark | Linear regression |
| Non-response at Previous sweeps (i.e. sweeps 2-9) |  |  | Yes vs no | Logistic regression |

**Section 3: MNAR analysis illustration with a (hypothetically valid) external benchmark.**

In this section we present an example of how our approach could be used if the missing data generating mechanism is suspected to be MNAR in the presence of an appropriate external benchmark. We conducted analysis on the BMI levels of individuals in BCS70 aged 34 from sweep 7 (age 34) in 2004. Multiple imputation with delta adjustment was employed, incorporating external information from the Health Survey for England (HSE) as a hypothetically valid external benchmark. We note that we use data from HSE for illustrative purposes only, as it is challenging for this to be considered a valid external benchmark for BCS70.

Initially, we computed the mean BMI levels in BCS70 separately for men and women born in England from a complete-case analysis. Subsequently, we employed multiple imputation, utilizing all predictors related to non-response at sweep 7 as auxiliary variables, along with observed BMI from all the other sweeps. Finally, we applied multiple imputation with delta adjustment[6,7], ensuring that the estimated BMI levels from the HSE[8] matched the mean BMI levels for men and women from the BCS70 dataset. The magnitude of the delta values also serves as an indicator of the plausibility of the MAR assumption: smaller delta values suggest the plausibility of MAR, while larger delta values favoured the MNAR assumption.

Specifically, we wanted to estimate the mean BMI levels for men and women in 2004, using BCS70 (sweep 7 - age 34). We calculated the mean BMI levels from an external source, Health Survey for England (HSE), and we wanted to observe if the estimates from BCS70, after implementing our missing data strategy would be close.

From HSE, we have that for men

mean(BMI aged 25-34) =26.28

mean(BMI aged 35-44) =27.80

and for women

mean(BMI aged 25-34) = 25.73

mean(BMI aged 35-44) = 26.82

To estimate the mean BMI levels from HSE, separately for men and women at age 34, we assumed that the mean levels would be a weighted average of the estimates of (men and women) aged 25-34 and 35-44 (this was the available granularity from HSE). More specifically, we estimated

mean(BMI at 34)= 0.6* mean(BMI aged 25-34) + 0.4* mean(BMI aged 35-44)

So, we have that

mean(BMI at 34 for men)= 26.89 kg/m^2^

mean(BMI at 34 for women)= 26.17 kg/m^2^

For the standard error of the above estimate we followed the following procedure. We knew only the standard errors $\mathrm{se}\left( \mathrm{BMI}_{men,25-34} \right)$ and $\mathrm{se}\left( \mathrm{BMI}_{men,35-44} \right)$ for men and women aged 25-34 and 35-44, so we first calculated the variance for BMI for men and women aged 25-34 and 35-44. For men, we calculated:

$$Var\left( \mathrm{BMI}_{men,25-34} \right)={\mathrm{se}\left( \mathrm{BMI}_{men,25-34} \right)}^{2}*n_{men, 25-34}$$

$$Var\left( \mathrm{BMI}_{men,35-44} \right)={\mathrm{se}\left( \mathrm{BMI}_{men,35-44} \right)}^{2}*n_{men, 35-44}$$

Then we estimated the standard error for men aged 34, $\mathrm{se}\left( \mathrm{BMI}_{men,34} \right)$, from the formula

$$\mathrm{se}\left( \mathrm{BMI}_{men,34} \right)=\sqrt{{0.6}^{2}*\frac{Var\left( \mathrm{BMI}_{men,25-34} \right)}{n_{men, 25-34}}+{0.4}^{2}*\frac{Var\left( \mathrm{BMI}_{men,35-44} \right)}{n_{men, 35-44}}}$$

We then performed the same procedure for women as well to estimate $\mathrm{se}\left( \mathrm{BMI}_{women,34} \right)$

To investigate the plausibility of the MNAR assumption compared to the MAR assumption when calculating mean BMI levels, we conducted an analysis on the BMI levels of individuals aged 34 from sweep 7 (age 34) in 2004. Multiple imputation with delta adjustment was employed, incorporating information from HSE as an external benchmark.

The delta values were calculated as follows: We denote as

***BMI_ref_1_*** *🡪 Average BMI in men from HSE*

***R_1_****🡪 Observed values of BMI in men from BCS70*

***BMI_ref_2_*** *🡪 Average BMI in women from HSE*

***R_2_****🡪 Observed values of BMI in women from BCS70*

**BMI_1_ 🡪** the estimated BMI in men in BCS70

**BMI_2_** 🡪 the estimated BMI in women in BCS70

**n_obs_1_ 🡪** the number of individuals with observed BMI values in men in BCS70

**n_obs_2_** 🡪 the number of individuals with observed BMI values in women in BCS70

**n_mis_1_ 🡪** the number of individuals with missing values on BMI in men in BCS70

**n_mis_2_ 🡪** the number of individuals with missing values on BMI in women in BCS70

We require that the average BMI estimates by sex from BCS70, after multiple imputation, would be the same as the corresponding estimates from HSE.

In other words, for i=1,…, n_1_(=${n\_obs}_{1}+{n\_mis}_{1}$) men and j=1,…, n_2_(=${n\_obs}_{2}+{n\_mis}_{2}$) women in BCS70 we set

$\frac{{n\_obs}_{1}*\left( \bar{{BMI}_{1i}*R_{1i}} \right)+{n\_mis}_{1}*\left( \bar{{BMI}_{1i}*\left( 1-R_{1i} \right)}*+\delta_{1} \right)}{{n\_obs}_{1}+{n\_mis}_{1}}={BMI\_ref}_{1}$ (1)

and

$\frac{{n\_obs}_{2}*\left( \bar{{BMI}_{2j}*R_{2j}} \right)+{n\_mis}_{2}*\left( \bar{{BMI}_{2j}*\left( 1-R_{2j} \right)}*+\delta_{2} \right)}{{n\_obs}_{2}+{n\_mis}_{2}}={BMI\_ref}_{2}$ (2)

If we solve the above equation (1) and (2) for $\delta_{1}$ (delta value for men) and $\delta_{2}$ (delta value for women) respectively, we obtain

$\delta_{1}=\frac{{BMI\_ref}_{1}*\left( {n\_obs}_{1}+{n\_mis}_{1} \right)-{n\_obs}_{1}*\left( \bar{{BMI}_{1i}*R_{1j}} \right)}{{n\_mis}_{1}}-\bar{{BMI}_{1i}*\left( 1-R_{1i} \right)}$ (3)

$\delta_{2}=\frac{{BMI\_ref}_{2}*\left( {n\_obs}_{2}+{n\_mis}_{2} \right)-{n\_obs}_{2}*\left( \bar{{BMI}_{2j}*R_{2j}} \right)}{{n\_mis}_{2}}-\bar{{BMI}_{2j}*\left( 1-R_{2j} \right)}$ (4)

We implemented the MI using the delta adjustment in 2 steps. First, we utilised the “mi chained” command in Stata to perform the imputation of BMI at age 34, using all the auxiliary variables for sweep 7 plus BMI at ages 10, 16, 26 and 30 and we omitted from the imputation models of all these variables BMI at age 34. We then added delta values ($\delta_{1}$ and $\delta_{2}$) separately for men and women as described above.

The mean BMI levels from HSE were 26.89 kg/m^2^ (95% CI: 26.59, 27.19) for men and 26.17 kg/m^2^ (95% CI: 25.81, 26.52) for women (Figure S2). The mean BMI levels from BCS70 from the complete case analysis of participants born in England were 26.51 kg/m^2^ (95% CI: 26.37, 26.65) for men and 25.13 kg/m^2^ (95% CI: 24.96, 25.29) for women, lower than those in HSE. If the HSE data are taken to be population-representative, this suggests that BCS70 sweep 7 respondents had lower BMI than would be expected (i.e. bias due to non-response). When we implemented multiple imputation, using all identified predictors of non-response at sweep 7 as auxiliary variables and all the previous BMI measurements from age 10 to age 30, the estimated BMI levels increased slightly to 26.64 kg/m^2^ (95% CI: 26.50, 26.77) for men and 25.35 kg/m^2^ (95% CI: 25.19, 25.50) for women, though remained below the HSE levels. The delta values that would be needed if using delta adjustment would be relatively small (0.49 kg/m^2^ in men and 1.80 kg/m^2^ in women), which could have been an indication that the MAR assumption was plausible. However, it is challenging to consider HSE a valid external benchmark, so our ability to draw such conclusions is limited, even when the delta values are small.

*Figure S2: Comparison of mean BMI levels in men (upper panel) and women (lower panel) at age 34 in 2004 estimated from Health Survey from England (HSE) and BCS 70. Multiple imputation using delta adjustment was used in BCS 70 to match mean estimates values of BCS 70 with HSE.*


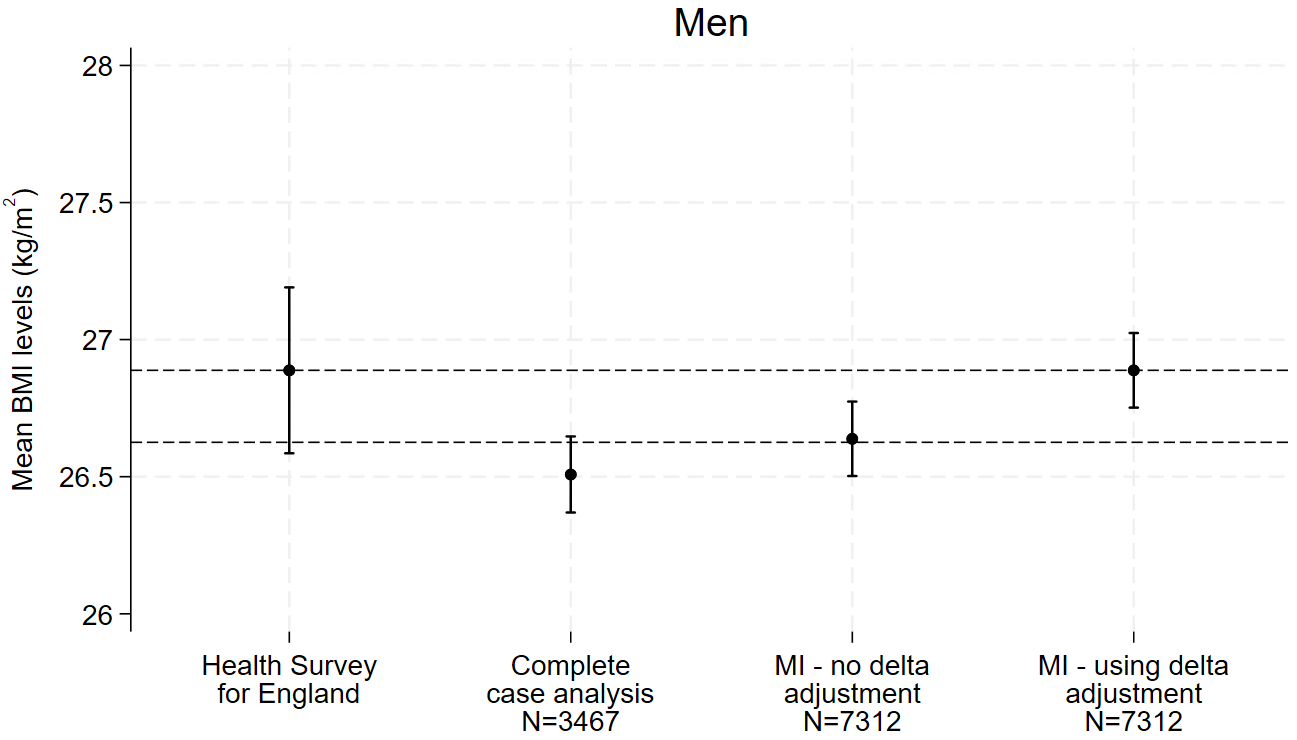

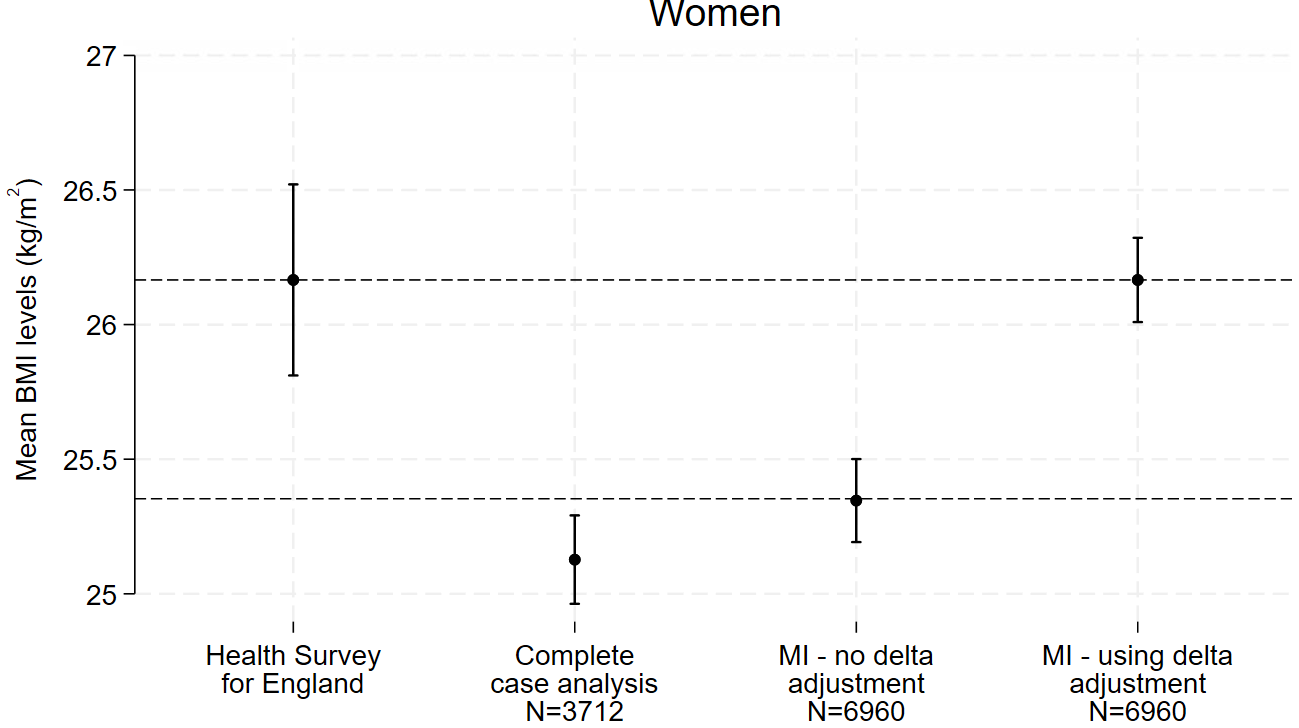


**Section 4: Similarities and differences between the NCDS and the BCS70 approach to derive the initial set of candidate variables as important predictors of non-response**

We use arbitrary thresholds in order to identify important predictors of non-response. We were largely based on the decisions made in the previous paper in NCDS, however we made some adjustments so that we avoid problems due to model overfitting or due to having too few individuals in stage 2. Then at Stage 3, we had the same criteria (p<0.001). Below we present in detail the similarities and differences between the NCDS[5] and the BCS70 approach to derive the initial set of candidate variables as important predictors of non-response

Our goal was to identify a manageable set of variables that can predict non-response, which researchers could consider incorporating into their analyses to better handle missing data. Specifically, we want a set that is large enough to enhance non-response handling but not so large that it becomes impractical (i.e. would result in too many additional parameters in a typical model).

**Table S21**. Deriving the initial set of candidate variables as important predictors of non-response

| Criteria | NCDS | BCS70 |
| --- | --- | --- |
| Routed variables | excluded “routed” variables (questions asked only of cohort members who gave a specific response to a previous question), used summary measures of scales rather the individual constituent items | The same |
| Binary variables with low prevalence | excluded all binary variables with prevalence < 1 % | The same |
| variables with item non-response | Exclude variables with item non-response > 50% | Exclude variables with item non-response > 40% |
| Stage 1 | p<0.05 | p<0.001 |
| Stage 2 | p<0.05 | p<0.05 |
| Stage 3 | p<0.001 | p<0.001 |
| Extra variables directly to stage 3 | no | sex, country of birth, participation in all previous sweeps and father’s socioeconomic status |

**References**

1. Zocchetti C, Consonni D, Bertazzi PA. Estimation of prevalence rate ratios from cross-sectional data. Int J Epidemiol 1995;24:1064–5
2. Royall RM. Model robust confidence intervals using maximum likelihood estimators. Int Stat Rev 1986;54:221–6.
3. Zou G. A modified poisson regression approach to prospective studies with binary data. Am J Epidemiol. 2004 Apr 1;159(7):702-6. doi: 10.1093/aje/kwh090. PMID: 15033648
4. Lachin JM. Biostatistical methods: the assessment of relative risks. New York, NY: Wiley-Interscience, 2000
5. Mostafa T, Narayanan M, Pongiglione B, Dodgeon B, Goodman A, Silverwood RJ, Ploubidis GB. Missing at random assumption made more plausible: evidence from the 1958 British birth cohort. J Clin Epidemiol. 2021;136:44-54
6. Leacy FP, Floyd S, Yates TA, White IR. Analyses of sensitivity to the missing-at-random assumption using multiple imputation with delta adjustment: application to a tuberculosis/HIV prevalence survey with incomplete HIV-status data. Am J Epidemiol. 2017;185:304–315
7. Katsoulis M, Lai AG, Diaz-Ordaz K, et al. Identifying adults at high-risk for change in weight and BMI in England: a longitudinal, large-scale, population-based cohort study using electronic health records. Lancet Diabetes Endocrinol. 2021;9(10)
8. NHS Digital Health Survey for England 2016: adult health trends. 2017. <https://files.digital.nhs.uk/publication/m/0/hse2016-adult-trends.pdf>
